# Factors affecting trust in healthcare among middle-aged to older Korean American women

**DOI:** 10.1186/s12905-018-0609-x

**Published:** 2018-06-22

**Authors:** Hye Chong Hong, Hyeonkyeong Lee, Eileen G. Collins, Chang Park, Lauretta Quinn, Carol Estwing Ferrans

**Affiliations:** 1 Mo-Im Kim Nursing Research Institute, College of Nursing, Yonsei University, 50-1 Yonsei-ro, Seodaemun-gu, Seoul, 03722 Korea; 2 College of Nursing, University of Illinois at Chicago, Chicago, IL USA

**Keywords:** Trust in healthcare providers, Trust in healthcare system, Discrimination in healthcare

## Abstract

**Background:**

Predictors of trust in healthcare providers and the healthcare system have never been studied in Korean Americans (KA) despite the fact that trust plays an important role in health behaviors. The purpose of this study is to examine factors influencing trust in the healthcare system and providers among KA women.

**Methods:**

Data were collected in 196 KA women examining the effects of perceived discrimination and trust on breast cancer screening in the Chicago metropolitan area. Path analysis was used to identify factors influencing trust in the healthcare system and providers.

**Results:**

Acculturation was positively related to trust in healthcare providers (*β* = .15, *p* =. 002), and discrimination in the healthcare system was inversely related to trust in healthcare providers (*β* = −.60, *p* <. 001). Length of stay in the US was inversely related to distrust in the healthcare system (*β* = −.14, *p* <. 001), and discrimination in healthcare was positively related to distrust in the healthcare system (*β* = .60, *p* <. 001). Trust in healthcare providers and distrust in the healthcare system were moderately correlated (*r* = .51, *p* < .001).

**Conclusion:**

Higher levels of acculturation and lower levels of perceived discrimination were identified as predictors of higher levels of trust in healthcare providers. A shorter stay in the US and higher levels of discrimination were identified as predictors of higher levels of distrust in the healthcare system. Perceived discrimination is a target for interventions to enhance trust in the healthcare system, and therefore reduce healthcare disparities in KAs.

## Background

Trust is an expectation that other parties will act in a patient’s best interest [1]. In healthcare, trust can be differentiated by the object of trust such as trust in the healthcare system and trust in healthcare providers [2–5]. Trust in the healthcare system is an important factor for promoting positive health behaviors, an indicator of high quality care and a crucial element of the patient-provider relationship [6–8]. Studies have identified that people have different levels of trust in their healthcare provider and the healthcare system [9–11]. Of concern is that minorities continue to report lower levels of trust and satisfaction toward their healthcare providers [12]. For example, African-Americans and Hispanics report consistently lower levels of trust compared to their White counterparts [13–15]. Recently, growing numbers of researchers have identified that trust has an important role in health behavior outcomes such as utilization of services, preventive screening, and adherence to medical advice [16–19].

Korean Americans (KAs) are one of the largest Asian subpopulations in United States [20, 21]. KAs consistently have lower preventive cancer screening rates including breast cancer and cervical cancer screening compared to other minorities and Asian Americans [22–24]. Several factors such as healthcare access, health beliefs, and cultural factors contribute to these low screening rates [24–42]. Despite interventions developed and delivered to KAs to increase cancer screening rates, cancer screening rates still remain low in KAs [21, 38].

In order to develop and implement effective interventions to increase trust in the healthcare system, and thus reduce healthcare disparities among KAs, it is vital to identify the factors contributing to low levels of trust. Given the positive relationship between trust in healthcare providers and preventive screening among other minority populations such as African Americans and Hispanics, identifying and addressing factors that undermine trust in healthcare providers and the healthcare system are important to promote positive screening behaviors. This analysis builds on our previous work [43] demonstrating that trust was an important factor influencing breast cancer screening in KA women. Our study was the first to identify that low trust levels in KA women are associated with lower rates of breast cancer screening [43]. The purpose of this analysis is to provide a detailed examination of the factors influencing trust in the healthcare system and healthcare providers among KA women.

### Predictors of trust

Perceived discrimination is identified as a significant predictor of trust or distrust in the healthcare system [13, 44]. Jacobs et al. [44] conducted 17 focus groups with African-American, Hispanic, and White participants to explore the factors contributing to trust and distrust in the healthcare system. In this study, both African American and Hispanics reported expectations of discrimination as determinants of distrust. Armstrong et al. [13] examined the relationship between racial discrimination and healthcare system distrust among African Americans and Whites. They reported prior experience of racial discrimination as a strong predictor for healthcare system distrust (OR 1.34, CI 1.23–1.46).

Other factors that predict trust or distrust in healthcare include race/ethnicity, age, education and income level. African Americans consistently reported lower levels of both trust in healthcare providers and the healthcare system [9, 10, 13, 14, 45–51]. Older age was consistently associated with lower levels of trust [8, 46, 50, 52, 53]. In contrast, in Chinese Americans, older age was associated with higher levels of trust in physicians (*r* = .16, *p* < .001) [12]. The relationship between education and trust is conflicting. Armstrong et al. [13], Armstrong et al. [14], and Halbert et al. [9] reported that less educated patients had lower levels of trust while Kayaniyil et al. [54], O’Malley et al. [19], and Simon et al. [12] identified a significant association between less education and higher levels of trust. Income and insurance status were inconsistently associated with trust. Armstrong et al. [14] reported that lower income was associated with lower levels of trust in physicians (measured by physician distrust scale) (OR .96, CI .94–.99) while Armstrong et al. [45] and Benjamin [52] found lower income was significantly associated with higher trust levels (measured by healthcare system distrust scale and trust in ones’ physician scale) (OR 2.52, CI 1.02–6.63; B = −.10, *p* < .01 respectively). Among Japanese Americans, higher levels of acculturation were associated with higher trust levels in physicians (*β* = .08, *p* < .001), and in Chinese Americans longer stay in US was related to higher levels of trust in healthcare providers [8, 12]. Cultural beliefs have not been examined as factors influencing trust levels. Cultural beliefs may be an important factor contributing to lower trust levels among KAs since the majority (78%) of KAs are first generation immigrants who may be influenced by traditional Korean values [55, 56]. Despite the fact that trust in plays an important role in health behavior outcomes, to our knowledge, our group is the first to study the predictors of trust in the healthcare system in KAs.

## Methods

### Design and sample

This study was a cross sectional survey that included 196 Korean American women. A detailed description of the methodology is reported elsewhere [43]. Participants were recruited from four Korean churches in the Chicago metropolitan area. Women were eligible if there were aged between 50 to 74 years, had no history of any type of cancer, and were able to read and understand either English or Korean. The questionnaires were provided in both English and Korean and, however all women answered in Korean. Each woman received 10 dollars when the questionnaire was completed. Data collection was started after approved by the Institutional Review Board at the University of Illinois at Chicago.

### Measures

#### Trust in healthcare providers

The Trust in Physician (TIP) questionnaire consists of 11 items that measures the level of interpersonal trust in the patient-physician relationship [57]. Each item is rated on 5-point Likert scale that ranges from strongly disagree (1) to strongly agree (5) with a total score ranges from 11 to 55. A higher score indicates a higher trust in healthcare providers. Internal consistency in the original scale was .90 and the Chinese version of TIP scale was .84 [12]. Construct validity was supported by a significant relationship between TIP and satisfaction with physicians in two studies (*r* = .62, *p* < 0.001; *r* = .73, *p* < 0.001) and TIP and desire for clinician’s control (*r* = .48, *p <* .001) [57, 58]. In this study sample, Cronbach’s alpha was 0.82 [43].

#### Trust in healthcare system

A revised Health Care System Distrust (HCSD) scale was developed by Shea et al. [59] to measure the system level of trust in healthcare. The HCSD includes 9 items that were developed from diverse racial/ethnic focus groups. The 9 items were rated on a 5-point Likert scale (strongly disagree, disagree, neither agree nor disagree, agree, or strongly agree), producing a total score ranging between 9 and 45. A higher score indicates higher distrust in healthcare system. The scale consists of two subscales (1) value distrust (5 items, Cronbach’s alpha = 0.73), and (2) competence distrust (4 items, Cronbach’s alpha = 0.77). Both validity and reliability were similar for African Americans and Whites [59, 60]. In this study sample, Cronbach’s alpha was 0.83 [43].

#### Perceived discrimination in healthcare

The modified version of Williams’ Everyday Discrimination Scale was developed by a Bird and Bogart to assess the level of perceived discrimination in healthcare settings [61, 62]. The Perceived Discrimination in Healthcare scale consists of 7-items and each item is scored on a 5-point Likert scale (1 = never, 2 = rarely, 3 = sometimes, 4 = most of the time, and 5 = always), producing a total score ranging from 7 to 35. The higher score means greater perceived discrimination. The Cronbach alpha was .60 in Latinas, .94 in American Indians, and .89 in African Americans. Factor analysis confirmed one factor solution [63–65]. Convergent validity was supported by a significant relationship between perceived discrimination in healthcare and societal discrimination (*r* = .51, *p* < .001), and perceived discrimination in healthcare and the overall African American Trust in Health Care scale with African Americans (*r* = .27, *p* = .02) [64]. In this study sample, the Cronbach’s alpha was 0.88.

#### Acculturation

A Short Acculturation Scale for Koreans (SAS-K) was used to assess the level of acculturation [66]. The 12-item SAS-K measures three dimensions of acculturation: (a) language use and preference at work, at home, and with friends; (b) language use and preference in media programs; and (c) preferred ethnicity of individuals in social relations. The items are rated on a 5-point Likert scale, ranging from 1 (only Korean) to 5 points (only English). The scores are averaged across items (range of scores is 1 through 5). A score closer to 1 indicates little acculturation and a score closer to 5 indicates high acculturation. The Cronbach’s alpha was 0.90 and each subscale ranged from 0.73 to 0.86 in this study sample. Construct validity was supported by moderate-to-strong correlations with age of arrival in the US (*r* = −.62, *p* < .001), length of residence (*r* = .51, *p* < .001), and English proficiency (*r* = .74, *p* < .001) [66].

#### Cultural beliefs

Cultural beliefs were assessed by the Cultural Beliefs Scale [67] which was designed to measure cultural beliefs contributing to later stage diagnosis of breast cancer among African American, Caucasian, and Hispanic women. This scale consists of 17 item measured in four different content areas (cultural beliefs related to breast lumps, self-help techniques, faith-based beliefs, and futility of treatment), answered in true or false response producing a total score ranging from 0 to 17. The higher score means a greater number of cultural myths are believed. In this study sample, the Cronbach’s alpha was 0.65.

#### Background variables

Socio-demographic variables such as age, length of stay in the US, education, marital status, income, employment, a health-related variable such as self-rated health (SRH), and access related variables such as insurance, and usual source of care (regular doctor) were collected.

### Translation process

The Trust in Physician (TIP), the revised Health Care System Distrust (HCSD), and the Perceived Discrimination in Healthcare scales were not used in the KA population previously. The following steps were used to confirm the accurate translation of the scales: (1) committee-based translation with 3 bilingual Korean PhD students, (2) expert review by a PhD professor, (3) pre-testing of translated scales using cognitive interviews to detect items and words that were not understood by the 10 Korean-American women between 50 and 74 years, and (4) review of the translated scales by an expert for final confirmation.

### Statistical analyses

All data analyses were conducted using Stata/IC version 12 and SPSS 24. Descriptive statistics such as means, standard deviations, frequencies, and percentages were used for data analysis. Bivariate analyses using t-tests and Pearson’s correlations were performed and significant variables (*p* < .01) were entered in the path analysis. Path analysis is a special case of structural equation model (SEM). Our model only consists of observed variables with assumption of no measurement error, thus path analysis was appropriate [68]. We have also tested the normality of dependent variables by Shapiro-Wilk, Shapiro-Francia (*p* > .10), and quantile-quantile (Q-Q) plot. We calculated the post-hoc power of the analysis using the value of the Root Mean Square Error of Approximation (RMSEA = < .05), degree of freedom (df = 17), sample size of 196, alpha level of 0.05 and the study yielded a power of 0.80 [69]. To assess model fit, several indices such as Tucker Lewis Index (TLI), RMSEA, and The Comparative Fit Index (CFI) were used**.**

## Results

A total of 196 Korean American women between 50 and 74 years old participated in this study. Tables 1 and 2 present the demographic data and associations between demographic variables and trust in healthcare. The sample consisted of middle-aged to older KA women who resided in the US for 2 to 5 years (Table 1). The majority of KA women sampled (80%) were naturalized immigrants and 75% were married. About 44% of KA women worked either part-time or full-time, and about 59% of KA women had college or higher education. Twenty-eight percent of the women had an income below 25,000 dollars. More than 90% had health insurance, and almost 85% of KA women had regular doctor or a regular place they could go for health care. About 78% of KA women rated their health as good or very good. Mean scores for major variables follow: acculturation 19.2 ± 7.2, discrimination in healthcare 13.2 ± 5.6, trust in healthcare providers 35.2 ± 7.4 and distrust in the healthcare system 25.9 ± 6.0.

**Table 1:** Descriptive Statistics (*N* = 196)

Variables	M	SD	Range
Age	62.73	6.78	50–74
Residency in US (yrs)	28.87	11.32	2–54
Acculturation	19.16	7.15	12–46
Discrimination in healthcare	13.20	5.55	7–28
Trust in healthcare providers	35.17	7.42	16–55
Distrust in healthcare system	25. 94	5.96	11–42

**Table 2:** Association between demographics and trust in healthcare (*N* = 196)

		Trust in healthcare providers		Distrust in healthcare system	
Variables	n (%)	M (SD)	p	M (SD)	p
Immigration status					
Immigrant (Naturalized)	156 (79.6)	36.2 (6.78)	<.001	25.2 (5.60)	<.001
Immigrant (Non-citizen)	34 (17.4)	31.2 (9.01)		28.9 (6.74)	
Marital status					
Married	147(75.0)	35.3 (6.76)	.769	25.8 (5.74)	.473
Not married	49(25.0)	34.9 (9.19)		26.5 (6.62)	
Employment					
Working	87 (44.4)	33.8(7.51))	.021	27.0 (6.21)	.029
Not working	109 (55.6)	36.3 (7.20)		25.1 (5.65)	
Education					
High school or less	80 (40.8)	34.8 (6.77)	.552	26.2 (5.61)	.561
College or higher	116 (59.2)	35.4 (7.85)		25.7 (6.21)	
Income (US$)					
< 25,000	46 (28.0)	36.0 (6.75)	.661	26.4 (5.58)	.361
> 25,000	118 (72.0)	35.5 (7.23)		25.5 (6.08)	
Insurance					
Yes	183 (93.4)	35.3 (7.40)	. 558	25.8 (6.01)	.182
No	13 (6.6)	34.0 (7.91)		28.1 (4.94)	
Regular doctor					
Yes	166 (84.7)	36.0 (7.20)	<.001	25.4 (5.75)	< .001
No	30 (15,3)	30.6 (7.06)		29.1 (6.22)	
Health status					
Good and above	153 (78.1)	35.8 (7.29)	.023	25.6 (5.82)	.128
Poor to fair	43 (21.9)	32.9 (7.48)		27.2 (6.35)	

Trust in healthcare providers in naturalized immigrants and those who were not working were significantly higher than non-citizen immigrants and those who were working (*p* < .001 and *p* = .021 respectively). Trust in healthcare providers was significantly higher in KA women who had a regular doctor or a usual place for healthcare and who rated their health as good and above than in women who did not have regular doctor or a usual place for healthcare and who rated their health as poor to fair (*p* < .001 and *p* = .023 respectively). Distrust in the healthcare system in naturalized immigrants and those who were not working were significantly lower than non-citizen immigrants and those who were working (*p* < .001 and *p* = .029 respectively). Distrust in healthcare system in KA women who had regular doctor or a usual place for healthcare was significantly lower than who did not have regular doctor or a usual place for healthcare (*p* < .001) (Table 2).

Table 3 represents the relationship among study variables. There was a moderately strong relationship between trust in healthcare providers and perceived discrimination in healthcare. Smaller relationships were identified between trust in healthcare providers and years of residency in the US, acculturation, and cultural beliefs. Similarly, moderately strong relationships were identified between distrust in the healthcare system and perceived discrimination in healthcare and trust in healthcare providers. Weaker relationships were identified with years of residency in the US and cultural beliefs (Table 3).

**Table 3:** Correlations between study variables (*N* = 196)

Measure	1	2	3	4	5	6	7
1. Age	−						
2. Residency in US	.44***	−					
3. Acculturation	.03	.24***	−				
4. Cultural beliefs	.06	−.16**	−.14**	−			
5. Discrimination in healthcare	−.11	−.24***	−.07	.39***	−		
6. Trust in Healthcare providers	.09	.29***	.22**	−.31***	−.67***	−	
7. Distrust in Healthcare system	−.09	−.32***	−.05	.30***	.65***	−.73***	−

*p* < .05**, *p* < 001***

**Fig. 1:**
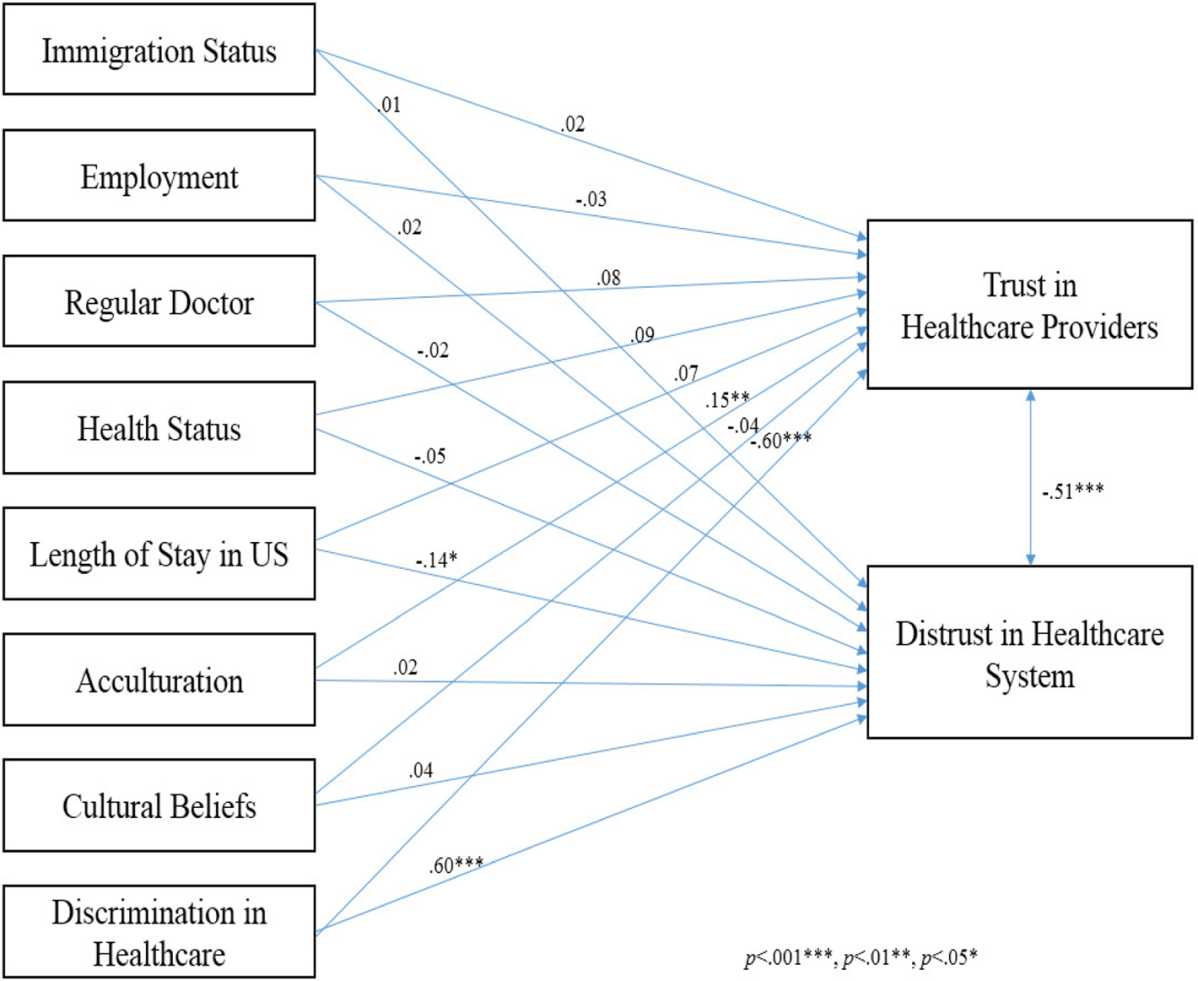
Structural relationships of factors contributing to trust in healthcare

In the path analysis model, acculturation was positively related to trust in healthcare providers (*β* = .15, *p* =. 002), and discrimination in healthcare was inversely related to trust in healthcare providers (*β* = −.60, *p* <. 001). Length of stay in the US was inversely related to distrust in healthcare system (*β* = −.14, *p* <. 001), and discrimination in healthcare was positively related to distrust in healthcare system (*β* = .60, *p* < .001). Immigration status, marital status, having a regular doctor or a usual place for healthcare, and cultural beliefs did not predict trust in healthcare providers and distrust in the healthcare system. Trust in healthcare providers and distrust in the healthcare system were moderately correlated (*r* = .51, *p* < .001) (Fig. 1). Fit indices reflected that the model was a good fit: CFI = 1.00, RMSEA <.001, TLI 1.00.

## Discussion

This is one of the first studies to examine factors influencing trust in healthcare among KAs. The major findings from this study were that (1) higher levels of acculturation and lower levels of perceived discrimination were identified as predictors of higher levels of trust in healthcare providers; (2) shorter stay in the US and higher levels of discrimination were identified as predictors of higher levels of distrust in the healthcare system; and (3) trust in healthcare providers and distrust in the healthcare system were highly correlated.

The mean score on the Trust in Healthcare Providers scale was lower in this sample (35.2 ± 7.4) than in 1111 out-patients with coronary artery disease (43.5 ± 6.3) [54], 119 newly diagnosed cancer patients (43.5 ± 7.3) [70], Chinese elderly (40.5 ± 6.2) [12], and in Taiwanese women (40.5 ± 5.7) [71]. The lower level of trust in healthcare providers may be explained by some of the demographic variables. For example, men tended to report higher trust levels in healthcare providers [52] and our sample included only women. Therefore, the mean score could have been lower than previous studies that included both women and men. Previous research has shown that higher education levels were associated with lower levels of trust in healthcare providers in the Chinese elderly (60 and older) [12]; our sample were highly educated women, however, education was not related to trust in healthcare in our bivariate analysis. The low level of trust could be explained by other factors that were found to be significant in this study which are discussed later.

In a study of 236 (144 African Americans, 92 whites) adults treated in primary care practices or the emergency department of a large, urban Mid-Atlantic health system, the overall mean score distrust in healthcare system was 25.2 (SD not reported) and was higher in African Americans (25.8) as compared to 24.1 in Whites [45]. In our study, the overall mean score of distrust in the healthcare system was 25.9 ± 6.0 which was similar to African Americans in the study of Armstrong et al. [45]. Other studies that focused on trust in the healthcare system were identified but used different measures. Moreover, there was no study assessing the level of distrust in healthcare system in Asian Americans or in Korean Americans, specifically.

Higher levels of acculturation predicted a higher level of trust in healthcare providers and a longer stay in the US was a significant predictor of lower levels of distrust in the healthcare system. The relationship between acculturation and trust in healthcare in Asian Americans is scarcely studied in literature since most studies focus on African Americans and Whites. Of the two studies that were completed with Asian Americans, Simon et al. [12], and Tarn et al. [8] reported those who lived longer in the US and were more acculturated showed higher levels of trust in healthcare providers in Chinese and Japanese Americans respectively which are similar to our findings. Longer stay in the US may indicate that a person is more acculturated, more familiar with the US healthcare system, and able to communicate better with healthcare providers.

Consistent with previous research, in our study perceived discrimination was a significant predictor of trust in healthcare providers and distrust in the healthcare system. Although a better understanding of perceived discrimination among KAs is needed, our sample reported higher perceived discrimination compared to other minorities such as African Americans and Hispanics. For example, although several investigators in previous studies did not report the mean values for the perceived discrimination in healthcare scale, Peek et al. [64] reported mean values of each item that ranged from 1.3±.6 to 2.0±.9 among 74 African Americans, with lower scores indicating lower perceived discrimination in the healthcare setting. In this study, the mean values for each specific item were higher and ranged from 1.7±1.0 to 2.2±1.0. These results may indicate that Korean American women perceive higher discrimination in the healthcare setting than even African Americans.

Major study findings suggest several areas for future research and implications for provider practice. The existing literature supports the idea that racial/ethnic discrimination and low trust in healthcare providers and the healthcare system are prevalent in minorities. However, there is a lack of national data comparing different racial/ethnic groups, which makes it difficult to identify trends among different minorities. Moreover, available studies use a variety of different instruments to measure perceived discrimination, trust in healthcare providers, and trust in healthcare systems, making comparisons difficult. Several authors have used only one or two questions, rather than established instruments. Therefore, in future studies the use of standardized reliable and validated instruments will contribute to a better understanding of experiences of discrimination and also those contributing to low trust at both interpersonal and system level. Doing so will help us understand how these phenomena work in healthcare settings and across different minorities while identifying targets for interventions.

As mentioned earlier, this study is the one of the first studies to assess and examine perceived discrimination in healthcare and trust in healthcare providers as well as trust in the healthcare system, and may not provide full explanation about their relationships. The retrospective findings of this study provide an overview of the factors influencing trust and thus further studies are needed using complex sampling methods and larger sample sizes to confirm the study findings and generalizability.

Although more studies are needed to confirm the study findings, reducing perceived discrimination is a target for interventions to enhance trust in healthcare, and therefore reduce healthcare disparities. The efforts to reduce discrimination in healthcare may include emphasizing professionalism among medical workforce [13] and competency training of healthcare providers [72, 73]. This effort may require system level policy to implement the continuous interventions or education to healthcare providers. The interventions developed for healthcare providers also need to be culturally sensitive and include specific needs of KA women.

There are several limitations to this study. We conveniently selected four Korean churches in Chicago and Metropolitan area for this study. The sample may have overrepresented women who are less acculturated since these women may be more affiliated with Korean culture than those who attend non-Korean churches. Also, the sample overrepresented Christians who may have a different level cultural beliefs compared to those who do not attend church at all. Moreover, almost 80% of women were naturalized immigrants and the mean length of stay in US was approximately 29 years. This may be due to the fact that our sample was women aged between 50 and 74 years. For future studies, a random cluster sampling of Korean churches and other Korean organizations including different socio-demographic characteristics would increase the generalizability of the study findings. The cross-sectional design of this study limits generalizability of the study findings and precedence among variables.

Trust could have been affected by other important factors. Although we tried to include and control for factors that were identified in previous literature, we could have missed some factors. For example, people with chronic diseases could have more chances to visit their doctors or healthcare providers and may had more chances for experiencing discrimination and lower or higher trusting relationship. Our study was a secondary analysis of data collected to examine the relationship between trust, discrimination, and breast cancer screening, and therefore was limited to data that were already collected. For the future studies, more variables may be included based on thorough literature review.

## Conclusions

Acculturation and perceived discrimination were significant predictors of trust in healthcare. Although acculturation is not amenable to change, perceived discrimination is a prime target for interventions to enhance trust in healthcare, and therefore reduce healthcare disparities. Reducing discrimination in healthcare will likely require multifaceted efforts, such as reiterating professionalism in medical workforce and cultural competency training of healthcare providers, particularly with the specific needs of KA in mind.

## Abbreviations

CFI: Comparative Fit index
HCSD: A revised Health Care System Distrust
KA: Korean American
RMSEA: Root Mean Square Error of Approximation
SAS-K: A short Acculturation Scale for Koreans
SRH: Self-Rated Health
TIP: Trust in Physician
TLI: Tucker Lewis Index
